# Aesthetic Leadership in Nursing: A Theoretical Proposal for Rehumanizing Care Delivery

**DOI:** 10.1111/nin.70034

**Published:** 2025-05-26

**Authors:** Jesús Tomás Monge Moreno

**Affiliations:** ^1^ University of the Balearic Islands Spain

**Keywords:** aesthetic leadership, healthcare transformation, humanized care, nursing leadership theory, nursing theory, rehumanization of healthcare

## Abstract

Aesthetic leadership in nursing constitutes a disruptive conceptual proposal that questions and redefines traditional leadership models within healthcare settings. This article develops an original theoretical framework that incorporates the aesthetic dimension as a structuring category of care, emphasizing the role of beauty, presence, and relational ethics in transforming healthcare delivery. Grounded in ontological, epistemological, ethical, and phenomenological dimensions, aesthetic leadership is framed as a praxis that transcends technical competence and operational management, aiming at transforming the caregiving experience and strengthening authentic bonds between nurses and patients. Based on a critical review of established nursing theories, including those proposed by Jean Watson, Imogene King, Betty Neuman, Madeleine Leininger, Patricia Benner, and Dorothy Johnson, a reconceptualization of leadership is proposed, integrating a holistic vision that articulates ethical, aesthetic, existential, and emotional dimensions in clinical practice. This theoretical model highlights the need to incorporate artistic, expressive, and symbolic resources into caregiving processes, with the purpose of enriching the caregiving experience, preserving human dignity, and promoting meaningful intersubjective relationships. Finally, it is argued that aesthetic leadership represents a pertinent pathway for the rehumanization of care in contemporary clinical contexts, proposing future research directions aimed at evaluating the impact of aesthetic practices on care quality, patient experience, and the professional well‐being of nurses.

## 1 Introduction

The practice of nursing faces innumerable challenges that transcend the application of clinical techniques and the management of healthcare resources (Hughes 2019; Aguirre Raya 2020; Elizalde Ordoñez et al. 2024). In a contemporary healthcare context, marked by technologization (Seibert et al. 2021; Pailaha 2023), bureaucratization (Inostroza‐Cisternas et al. 2024), and increasing caregiving pressure (Rosa et al. 2020; Kelly et al. 2021; Ballesteros‐Barrado 2023), significant concerns emerge regarding the dehumanization of care (Melita et al. 2021; Caicedo‐Lucas et al. 2023; García‐Uribe et al. 2024). This scenario complicates the relationship between healthcare professionals and patients (Labrague et al. 2018; Membrive‐Jiménez et al. 2020), generating a tangible risk of reducing the individual to an object of intervention, to the detriment of their status as an active and dignified subject. In light of this reality, nursing is called upon to revitalize its humanizing essence through leadership models that integrate ethics, aesthetics, and commitment to care.

Nursing leadership has been the subject of numerous theoretical and practical reflections over the past decades (Leclerc et al. 2020; Abraham et al. 2021; Northouse 2025). Models such as transformational and congruent leadership have sought to respond to this need, promoting motivation and professional involvement in patient care. However, their theoretical and practical limitations, especially in complex clinical settings, have highlighted the need for a more holistic approach centered on the art of caring (Monge‐Moreno 2023).

In response to the limitations of previous models, aesthetic leadership (Hansen et al. 2007; Gahlot and Bathla 2021; Monge‐Moreno 2023) emerges as an innovative and necessary proposal, a new approach that unites humanism, ethics, and an integrative vision of nursing practice. Unlike other paradigms centered on the individual traits of the leader, aesthetic leadership is based on the perception and recognition of others for the ability to positively influence the environment, lead with compassion, justice, and truth, and serve as a model in daily practice, positioning the leader as a moral and professional reference who inspires trust and adherence. Rooted in values (Palacios and Pabón 2019; Navarrete Romero et al. 2023; Amezcua 2024) such as justice, truth, and respect for human dignity, this model places the patient at the center of the caregiving process, promoting comprehensive care that responds not only to biological needs but also to the psychological, social, and spiritual dimensions of the human being (Larico Calla and Mamani Quispe 2020; Sălcudean 2022).

Aesthetic leadership (Farasat et al. 2024) is not a form of imposed authority but emanates from professional competence, integrity, and the leader's ability to accompany and guide with empathy and sensitivity. Aesthetic leaders do not self‐designate; they emerge from the recognition of their peers and the firm moral purpose that guides their practice (Hansen et al. 2007; Monge‐Moreno 2023). This leadership manifests in three fundamental dimensions: scientific‐technical, affective, and ethical. The harmonious integration of these dimensions ensures care based on knowledge and professional excellence but also on compassion, accompaniment, and respect for the individuality of each patient (Gahlot and Bathla 2021).

This leadership model is based on a relational ontology and an aesthetic epistemology, which recognize the complexity, subjectivity, and emotionality inherent in nursing care. This perspective allows for an understanding of caregiving phenomena beyond figures and clinical indicators, attending to the meanings, atmospheres, and shared experiences that shape daily clinical practice. Unlike the functionalist and quantitative approaches predominant in healthcare management, aesthetic leadership is concerned not only with visible actions but also with the implicit, affective, and symbolic dimensions that shape the nurse‐patient relationship and caregiving environments (Hansen et al. 2007; Monge‐Moreno 2023).

Aesthetic leadership focuses on the relationship between the leader and their collaborators, as well as on the deep connection with the patient (Magalhães 2024). This relational approach, based, among others, on intentional presence, recognition of the other, and clinical‐ethical decision‐making, allows for the creation of an environment of trust and collaboration that fosters care quality and the humanization of assistance (Monge‐Moreno 2023).

The application of aesthetic leadership in daily nursing practice has profound implications for the rehumanization of care (Poursaadat et al. 2024). From this perspective, the exercise of leadership aims not only to achieve optimal clinical results but also to accompany the patient and their loved ones through the health‐illness process, transforming nursing practice into a sublime art.

However, aesthetic leadership also faces challenges and moments of uncertainty, especially in clinical‐ethical decision‐making, where conflicts may arise between different stances and priorities (Latifi et al. 2023; Hager et al. 2024). Recognizing these challenges as an inherent part of leadership strengthens the necessary competencies to address them with prudence, temperance, and professional maturity, favoring reflective and conscious management that promotes quality and safety in care.

In conclusion, aesthetic leadership in nursing constitutes an innovative paradigm for the rehumanization of care. By harmonizing technique with the art of the profession, it fosters aesthetic environments as therapeutic and dignifying spaces, emphasizing the human dimension of those involved in the clinical relationship. This approach not only drives the professional development of healthcare teams but also transforms the experience of patients and their loved ones, promoting compassionate, dignified, and ethical care. In this way, aesthetic leadership emerges as a necessary response to contemporary healthcare system challenges and as a driver of change toward more human‐centered care committed to the essence of nursing.

## 2 Conceptual Framework

Through a comparative and critical analysis, aesthetic leadership integrates, redefines, and expands essential elements of the theories of Watson, King, Neuman, Leininger, Benner, and Johnson. The result is a leadership paradigm that combines aesthetic sensitivity, ethical depth, and relational creativity. This model not only aims to enhance the technical quality of care but also seeks to transform healthcare environments by fostering comprehensive care, cultural inclusion, continuous professional development, and emotional well‐being for both patients and nurses. Thus, aesthetic leadership offers a holistic vision of care that transcends mere technical management, promoting a more humane, ethical, and comprehensive caregiving experience.

### 2.1 Jean Watson: Ontological Foundation and the Limits of Transpersonal Care

Jean Watson's Human Caring Theory Watson (1997) provides a fundamental basis for aesthetic leadership by conceptualizing care as a transpersonal act that transforms both the caregiver and the recipient of care. Watson emphasizes the importance of authentic presence, compassion, and spirituality in the nurse‐patient relationship, placing human dignity at the core of caregiving praxis. This approach aligns with the principles of aesthetic leadership, which also emphasizes the humanity of the encounter and the transformation that emerges from compassionate interaction.

However, aesthetic leadership goes beyond the individual dimension of the nurse‐patient relationship. While Watson (2005, 2007) highlights the transpersonal bond between the individual and care, aesthetic leadership introduces a broader vision, incorporating institutional structures, collective contexts, and cultural languages as realities where care also manifests. In this sense, aesthetic leadership transcends the interiority of the encounter, creating environments, rituals, narratives, and symbols that contribute to the meaning of care in a visible and structural manner.

### 2.2 Imogene King and Betty Neuman: From Systemic Organization to the Aesthetic Atmosphere of Care

Imogene King's Goal Attainment Theory and Betty Neuman's Systems Model offer fundamental frameworks for understanding the role of care environments in the patient experience, yet they approach this from distinct perspectives, which resonate with the proposal of aesthetic leadership.

Imogene King (1981), by centering her theory on goal attainment through clear communication, goal negotiation, and dynamic interaction between nurse and patient, offers a vision focused on the interpersonal relationship as a continuous and fluid process. This theory proposes that care is an interactive phenomenon, based on the joint construction of goals between the patient and the nurse, with both parties being active participants in the caregiving process. King (1981) emphasizes the importance of open communication, negotiation, and joint decision‐making, where the nurse facilitates the process by helping the patient identify needs and establish clear objectives. This relational approach underscores a horizontal dynamic, where care is enacted through dynamic, bidirectional interaction.

Aesthetic leadership draws upon and expands this dimension of King's dialogical relationship, projecting it into a richer and more complex dimension by integrating the aesthetic‐emotional component of care. While King's theory focuses on goal attainment as the ultimate purpose of the caregiving process, aesthetic leadership introduces a purpose beyond measurable outcomes. Through the creation of aesthetically rich spaces, emotionally welcoming environments, and sensory‐pleasing experiences, aesthetic leadership seeks to foster an experience that is not limited to solving specific problems but aims to create meaningful and transformative experiences for the patient. This approach recognizes that the interaction, although fundamental, is not restricted to goal attainment or achieving tangible results but also encompasses the emotional and existential dimension of care, where the act of caring becomes a process of humanization through aesthetics.

Betty Neuman's theory situates care within a holistic and preventive perspective, focusing on protecting the patient from stressors that threaten their equilibrium. Her model, based on systems theory (Neuman 1974, 1982), views the human being as an open system in constant interaction with the environment, promoting interventions that strengthen the patient's adaptive capacities, fostering functional stability at the physical, psychological, sociocultural, spiritual, and developmental levels.

Aesthetic leadership adopts this comprehensive understanding of care but extends it beyond the homeostatic logic and rational control of variables. Instead of limiting the caregiving environment to a field of technical intervention on stressors, aesthetic leadership redefines it as a symbolic, relational, and sensory space that actively participates in the subjective configuration of the patient's experience. From this perspective, well‐being depends not only on the absence of stressors but also on the symbolic and aesthetic quality of the environment in which the experience of illness unfolds. This conceptual expansion allows for the integration of not only prevention and control but also creativity, intentional presence, and aesthetic sensitivity as legitimate and necessary foundations of leadership in contemporary healthcare contexts. Furthermore, aesthetic leadership underscores that the environment not only influences the patient as an external agent but also constitutes a space of meaning, where senses, emotions, and connections acquire an aesthetic and transformative dimension, referred to as the *aesthetic atmosphere of care*. In this space, harmony, beauty, and sensitivity emerge as therapeutic means capable of transfiguring the illness process into a meaningful experience.

### 2.3 Madeleine Leininger: Aesthetic Pluralism and Cultural Recognition

Madeleine Leininger's Theory of Transcultural Nursing (Leininger 1991, 1995, 2002) is essential for an aesthetic approach to care that recognizes and values cultural differences. Leininger postulates that every act of care is mediated by cultural background and can only be legitimate if it respects the beliefs and values of the patient. Aesthetic leadership builds upon this premise but goes further, proposing that there is not a single aesthetic of care but multiple forms of beauty and harmony that vary according to the cultural traditions of the individuals involved. Thus, aesthetic leadership advocates for an *aesthetic co‐creation* in the caregiving process.

This approach not only includes the cultural adaptation of clinical interventions but also encompasses the design of the environment and the creation of experiences that are aesthetically congruent with the values and customs of the patient. Aesthetic leadership promotes care that respects cultural diversity through a caregiving design that is both clinically appropriate and aesthetically satisfying, contributing to a more integral and holistic caregiving experience, that is, from cultural competence, sensitivity, recognition, and otherness.

### 2.4 Patricia Benner: Embodied Wisdom and Intuition

Patricia Benner's (1984) theory on the development of clinical judgment through experience (from novice to expert) validates tacit, sensory, and intuitive knowledge, which is crucial for aesthetic leadership. Benner emphasizes that nursing knowledge is situational, embodied, and relational, highlighting the importance of bodily sensitivity, lived experience (or situated practice), and pre‐reflective understanding. These elements resonate deeply with the principles of aesthetic leadership, which emphasize sensory and emotional aspects in caregiving practice.

However, aesthetic leadership introduces a key distinction: it values experience not only as a means of clinical decision‐making but also as a source of ethical and aesthetic sensitivity. Instead of limiting itself to technical expertise, the aesthetic leader also becomes an attentive observer of existential nuances, emotional atmospheres, and gestures, creating a transformative presence that fosters an affective resonance with the environment. In this sense, leading aesthetically also involves educating the gaze, the ear, and the word to create an affective and aesthetic resonance with the environment. Thus, aesthetic leadership recognizes experience not only as accumulation but also as the cultivation of artistic and relational sensitivity toward the other.

### 2.5 Dorothy Johnson: From Behavioral Equilibrium to the Symbolic Choreography of Care

Dorothy Johnson's Behavioral System Model (Johnson 1980) focused on regulating the patient's behavior patterns to restore equilibrium, stability, and order within the caregiving process, is also relevant to aesthetic leadership. However, the latter expands this perspective by addressing it from a symbolic and emotional dimension. In this context, behavior is not understood merely as a biological phenomenon but as a subjective and ritualized expression that must be embraced with aesthetic and emotional sensitivity.

Beyond functionality, aesthetic leadership recognizes the transformative potential of forms: the way news is delivered, the bed is made, or a hand is held can become an artistic and symbolic act of care. Unlike Johnson's model, which prioritizes the restoration of stability and order, aesthetic leadership also proposes generating new meanings from human disorder, caused by illness and suffering, among other factors. Thus, the way each action is performed, from a gesture to a conversation, can be transformed into an aesthetic practice with the power to deeply humanize care, both for the patient and the nurse.

In this regard, one of the key contributions of aesthetic leadership to Johnson's model is the *symbolic choreography of care*. This expression refers to the conscious and harmonious use of gestures, postures, words, and silences in the caregiving process. Each action, no matter how small, becomes a movement within a symbolic dance that conveys more than a mere response to a physiological or emotional need. It is a set of interactions where the intentional presence of care becomes something visible, transformative, and profoundly meaningful.

## 3 Aims

The general objective of this scientific article is to develop and substantiate an innovative theory on aesthetic leadership in nursing, integrating the aesthetic, emotional, and ethical dimensions of leadership to promote the rehumanization of care and professional practices within the healthcare setting. This study seeks to critically analyze existing leadership models, identify their limitations, and propose an approach that transforms the nurse‐patient relationship, optimizes the quality of healthcare, and fosters the improvement of health outcomes through a more humane, ethical, compassionate, comprehensive, and aesthetically‐oriented caregiving practice.

## 4 Methodology

### 4.1 Study Design

This study adopts a systematic literature review design with a theoretical‐constructivist approach, aimed at conducting a critical analysis and evaluation of the existing scientific findings on aesthetic leadership in nursing and its contribution to the humanization of care. This approach not only synthesizes the available evidence but also identifies conceptual gaps, theoretical tensions, and methodological limitations in the leadership models traditionally applied to nursing.

Through this methodology, the study seeks to comprehensively compare existing leadership models, confronting them with the principles and foundations of aesthetic leadership. This critical comparison allows for the identification of strengths and weaknesses in the predominant approaches, to substantiate the need for a new perspective.

The design of this study is oriented towards detecting patterns, trends, convergences, and gaps in the scientific literature, providing a solid epistemological and methodological foundation for the development and substantiation of an innovative theory on aesthetic leadership in nursing. In this way, the aim is to provide a theoretical framework that transforms the nurse‐patient relationship, optimizes the quality of healthcare, and promotes a more humane, ethical, compassionate, and aesthetically‐oriented professional practice.

### 4.2 Search Methods

#### 4.2.1 Data Sources

Searches were conducted in the electronic databases PubMed, Web of Science, CINAHL, and PsycInfo, using structured combinations of search terms that included MeSH descriptors and key terms related to nursing leadership, the humanization of care, patient experience, and other relevant concepts (Supporting Information S1). After conducting a pilot phase, a detailed search strategy was developed to identify relevant literature published without temporal restrictions. Searches were performed in both English and Spanish, and were executed over a 2‐week period starting from March 14, 2025.

#### 4.2.2 Inclusion and Exclusion Criteria

Studies that explicitly addressed leadership in the field of nursing, research potentially related to the aesthetics of care as a fundamental dimension in professional nursing practice contextualized within nursing leadership, and its impact on the humanization of care and caregiving relationships were included in the review. Clinical studies, meta‐analyses, reviews, systematic reviews, observational studies, and historical studies involving nurses in healthcare settings were selected.

Only peer‐reviewed publications in English and Spanish, with no temporal restriction on the publication date, were considered. Descriptive, qualitative, and quantitative studies that assessed the impact of leadership in nursing practice, with a particular focus on aesthetic leadership, humanization of care, and the relational experience between nurse and patient in the caregiving context, were included.

In contrast, studies addressing these topics marginally or tangentially were excluded. Also excluded were commentaries, editorials, letters to the editor, book chapters, unpublished theses, and opinion articles. Additionally, research lacking a clear theoretical framework or failing to present solid empirical evidence was excluded. To avoid duplication, repeated documents identified in the searched databases were removed.

#### 4.2.3 Study Selection and Data Extraction

In accordance with the PRISMA method (Moher et al. 2009), the total number of articles obtained through the previously defined search strategy in the PubMed, Web of Science, CINAHL, and PsycInfo databases was first determined. Subsequently, the inclusion and exclusion criteria were applied to refine the selection of studies relevant for analysis.

The initial screening of abstracts was conducted independently by the author of this study, who assessed the eligibility of the articles. During this phase, studies that were duplicates, did not meet the study objectives, or presented methodological limitations incompatible with the requirements of the systematic review were excluded.

Potentially relevant articles underwent a second phase of evaluation, during which the full text was reviewed to confirm its compliance with the established inclusion and exclusion criteria. Finally, pertinent data were extracted and organized in an Excel table, gathering information about the study characteristics, participant data, key findings, and corresponding recommendations. A total of 25 studies were included in the systematic review (Supporting Information S2).

## 5 Results

The critical analysis of the selected articles reveals a progressive and multifactorial evolution in the conceptualizations of leadership in nursing, characterized by a transition from traditional authoritarian, bureaucratic, or functional models to more participatory, relational, ethical, and transformational approaches. This transformation is driven by the increasing complexity of contemporary healthcare systems, organizational reforms, and a growing sensitivity toward the subjective experience of the patient and the well‐being of healthcare professionals.

Throughout the reviewed period (1994–2025), there is evidence of a gradual reconfiguration of nursing leadership frameworks, with a growing emphasis on dimensions such as relationality, authenticity, compassion, moral coherence, and commitment to the humanization of care. However, there is a widespread absence of explicit theorization of the aesthetic dimension of leadership, despite the fact that several of the models analyzed incorporate related elements such as creativity, compassionate care, emotional reflexivity, human dignity, and the symbolism of the therapeutic environment.

Based on the thematic analysis, five major categories were identified that structure contemporary conceptions of leadership in nursing.

### 5.1 Organizational and Cultural Transformation

This category includes contributions that link leadership with structural reform, the humanization of care environments, and the improvement of organizational outcomes (Klakovich 1994; Kitson 2001; Jackson et al. 2009; Leigh et al. 2015; McCay et al. 2018; Moraca et al. 2024). Leadership is presented as a driver of institutional change, a promoter of professional empowerment, and a guarantor of healthy environments. Leadership styles such as transformational, clinical, empowering, and knowledge‐centered nursing leadership (Kitson 2001; Jackson et al. 2009; Leigh et al. 2015) are identified, alongside critiques of bureaucratic, technocratic, and hierarchical models in favor of participatory, connective, and dialogue‐oriented paradigms (Klakovich 1994). Key issues such as crisis management, the culture of error, staff retention, and patient safety are also addressed (McCay et al. 2018; Harrington 2021; Moraca et al. 2024).

#### 5.1.1 Ethics and Care Values

In this category, leadership is conceived as ethical praxis and an ontological commitment to human dignity (Kowalski and Yoder‐Wise 2004; Gustafsson and Stenberg 2017; Esmaelzadeh et al. 2017; Honkavuo et al. 2018; Markey et al. 2022; Solbakken et al. 2022; Almutairi et al. 2025). The analyzed proposals promote ethical, servant, dialogical leadership focused on human values, where the leader is viewed as a moral agent embodying integrity, coherence, empathy, and compassion. This category is structured around the ethics of care, the promotion of organizational moral climate, cultural equity, and decision‐making contextualized in the complexity of healthcare settings (Esmaelzadeh et al. 2017; Markey et al. 2022).

### 5.2 Relationality and Human Connection

Here, leadership is interpreted as a relational competency that enhances trust, authentic communication, active listening, meaningful presence, and affectivity (Wong and Cummings 2007; Pipe 2008; McCloughen et al. 2009; Cardiff et al. 2018; Papadopoulos et al. 2022; Hult et al. 2023). This category is grounded in relational, resonant, compassionate, democratic, authentic, and service‐oriented leadership styles, with an emphasis on accompaniment, mentorship, and the promotion of staff well‐being and care quality. Leadership is seen as an interpersonal practice focused on human connection and the co‐construction of meanings in the caregiving experience.

### 5.3 Personal, Spiritual, and Existential Development of the Leader

This category identifies introspective, reflective, and compassionate leadership that incorporates spirituality, self‐care, cultivating inner silence, and personal growth as the foundation of ethical‐aesthetic leadership (Wong and Cummings 2007; Pipe 2008; Rudolfsson and Flensner 2012; Honkavuo et al. 2018; Ribeiro et al. 2021). Spiritual, authentic leadership, rooted in caritas processes, is linked to a hermeneutic understanding of human suffering and caregiving as an experience of meaning. This approach emphasizes the need for leaders who develop inwardly to be able to accompany others existentially and emotionally, fostering a humanly sustainable leadership.

### 5.4 Metaphors of Care and Organizational Symbolism

Finally, this category explores leadership as an aesthetic, symbolic, and artistic experience capable of transforming caregiving environments through gestures, presence, narratives, and objects imbued with meaning (Rudolfsson and Flensner 2012; Cardiff et al. 2018; Solbakken et al. 2018; Anglin et al. 2020; Salminen‐Tuomaala and Seppälä 2022). There is a theoretical openness towards leadership models sensitive to the aesthetic, emotional, and symbolic dimensions, where art, poetry, metaphors of care, and empathetic presence create a new way of leading from the imperfect beauty of human fragility. Although these proposals have not yet developed a structured institutional framework, they open a fertile field for rethinking nursing leadership as an aesthetic practice applied to organizational culture, space design, and the symbolic transformation of the caregiving environment.

## 6 Discussion

The findings from this systematic review indicate that the approach to leadership in nursing does not follow a linear, coherent, or progressive evolution; rather, it is configured as a constellation of fragmented proposals that only partially engage with each other, failing to resolve persistent epistemic, ontological, and political tensions within the disciplinary discourse. In this context, it is significant that none of the analyzed leadership categories explicitly incorporates the aesthetic dimension as a core component of contemporary nursing practice. This omission is not merely a conceptual gap but rather a symptom of a positivist and technocratic rationality that has colonized the disciplinary field, displacing sensitive knowledge, emotionality, the poetics of the environment (understood as the symbolic, aesthetic, and emotional capacity of a physical or relational space to generate meaning, affective resonance, and significant experience for those who inhabit it), as well as the existential dimension of care.

From a critical perspective, the coincidences, omissions, and tensions identified allow for the reconstruction of a theoretical map in which nursing leadership has prioritized categories such as relational management, professional empowerment, and the humanization of care, but has lacked an epistemological‐operational conceptualization of aesthetics as an organizing principle of power relations, clinical spaces, and the symbolic and emotional interactions between patients, nurses, multidisciplinary teams, affective circles, and communities.

It is important to highlight that the nursing leadership models analyzed agree on the need for an ethical, humanized praxis focused on patient well‐being, prioritizing values such as empathy, communication, and relational care (Kitson 2001; Kowalski and Yoder‐Wise 2004; Wong and Cummings 2007; Pipe 2008; Esmaelzadeh et al. 2017; Cardiff et al. 2018; McCay et al. 2018). Additionally, some models incorporate collaborative approaches, participatory leadership, and shared decision‐making (Klakovich 1994; Jackson et al. 2009; Leigh et al. 2015), reinforcing a focus on horizontal care and co‐construction. Transformational leadership, relational leadership, and ethical leadership agree in placing the nurse‐patient relationship and the emotional well‐being of the team at the center of professional practice, proposing leadership styles based on trust, cooperation, and active participation (Wong and Cummings 2007; Cardiff et al. 2018; Salminen‐Tuomaala and Seppälä 2022; Hult et al. 2023). Meanwhile, humanized and existential‐spiritual leadership models prioritize integrating the spiritual, emotional, and relational dimensions of care, offering ethical‐existential perspectives, and in some cases, opening spaces for implicit aesthetic reflection (Rudolfsson and Flensner 2012; Solbakken et al. 2018; Honkavuo et al. 2018; Harrington 2021; Markey et al. 2022). However, despite these convergences, none of these proposals incorporate aesthetics as an epistemological, structuring category of relationships, spaces, and experiences in the caregiving environment, leaving this dimension either implicit or subordinated to ethical, relational, and existential frameworks.

Systematically, all the nursing leadership models reviewed omit an explicit conceptualization of aesthetics as a constitutive category of leadership and nursing practice. Transformational leadership prioritizes rational decision‐making, professional empowerment, and value management (Klakovich 1994; Jackson et al. 2009), while relational leadership focuses on communication, active participation, and strengthening team cohesion (Wong and Cummings 2007; Hult et al. 2023). However, none of these models address the aesthetic, symbolic, or sensory experience of care as a structural component of leadership. Similarly, ethical leadership and authentic leadership, while promoting integrity, moral coherence, and reflective dialogue (Esmaelzadeh et al. 2017; McCay et al. 2018; Markey et al. 2022), lack a reflective approach to the environment from an aesthetic perspective. Relational and humanized models highlight the quality of interpersonal relationships and emotional accompaniment (Cardiff et al. 2018; Solbakken et al. 2018), but do not explore the aesthetic configuration of the clinical space or the symbolic dimension of therapeutic atmospheres. Even when spatial metaphors such as the “House of Care” or the “leader's secret room” are introduced (Solbakken et al. 2018), the aesthetic analysis remains implicit or subordinated to the ethical‐relational plane. Similarly, existential‐spiritual leadership incorporates suffering, dignity, and philosophical reflection as components of care (Rudolfsson and Flensner 2012; Honkavuo et al. 2018), but does not articulate an aesthetics of leadership nor propose symbolic or creative mechanisms to reframe clinical environments. Harrington (2021) and Markey et al. (2022), while highlighting the emotional and moral impact of leadership, do not address beauty, creativity, or aesthetic sensitivity as organizing principles of care. This widespread omission represents a significant epistemic gap, as it neglects the structuring role of the aesthetic dimension at three fundamental levels of the healthcare environment: first, in the configuration of subjectivities, i.e., the way in which patients, nurses, multidisciplinary teams, affective circles, and communities construct their identities and emotional experiences in caregiving contexts; second, in the structuring and reproduction of power relations within teams and healthcare organizations; and third, in the generation of sensitive, symbolic, and emotionally meaningful experiences that provide meaning to the acts of caregiving and being cared for (Gustafsson and Stenberg 2017; Ribeiro et al. 2021; Solbakken et al. 2022). In this framework, aesthetic leadership cannot be understood merely as a conceptual innovation within the disciplinary discourse but must be interpreted as an ontological and political necessity: ontological, in that it allows for the reconfiguration of being‐in‐care from a sensitive and embodied rationality; and political, in that it critically challenges institutional structures, power dynamics, and symbolic forms that organize clinical experience. Against conventional models that, while promoting humanization processes, fail to sensitively transform the caregiving experience, aesthetic leadership introduces an alternative logic based on the sensitive, symbolic, and beautiful as constitutive categories of nursing practice.

The identified tensions are related to the difficulty of nursing leadership models in coherently and articulately integrating the rational, ethical, emotional, spiritual, and aesthetic dimensions into an integral praxis. Transformational, relational, and ethical models tend to prioritize rationalistic and deontological categories (Wong and Cummings 2007; Jackson et al. 2009; Esmaelzadeh et al. 2017; Salminen‐Tuomaala and Seppälä 2022), sidelining the sensitive, symbolic, and aesthetic knowledge that shape the act of caring. This reductionist tendency reinforces a functional view of leadership that, while improving the organization of work, fails to create emotionally inhabitable atmospheres or aesthetically meaningful settings. In contrast, models such as humanized leadership and existential‐spiritual leadership partially reclaim the emotional and spiritual dimensions of care (Rudolfsson and Flensner 2012; Solbakken et al. 2018; Honkavuo et al. 2018). However, these approaches do not transcend the individualist ontological framework nor structurally challenge the aesthetic, symbolic, and political logics that configure the clinical environment. Authors such as McCloughen et al. (2009) acknowledge the importance of human connection and mentorship in leadership development but do not delve into the role of aesthetics as a transformative catalyst for relationships or the environment. On the other hand, Moraca et al. (2024) and Almutairi et al. (2025) introduce notions such as open communication, relational courage, authenticity, and team emotional well‐being, which are fundamental to an aesthetic praxis of leadership. However, both proposals maintain a focus on organizational culture, without articulating an aesthetics of spaces, gestures, or therapeutic atmospheres. This limits not only the humanization of leadership but also its capacity to resonate with the lived experience of patients and nurses on the sensory, emotional, and symbolic level. Papadopoulos et al. (2022), from the perspective of compassionate leadership, offer a valuable opening toward recognizing human suffering as an ethical and affective experience, integrating cultural competence as an expansion of the symbolic horizon of care. However, they do not formalize aesthetics as a category nor explore the role of beauty, harmony, or creativity in generating humanized care spaces. This fragmentation highlights the need for an integrative proposal that overcomes the dichotomies of reason‐emotion, technique‐humanism, and ethics‐aesthetics. In this framework, aesthetic leadership emerges as a new epistemological, ethical, and political paradigm, capable of sensitively articulating the different dimensions of care into an embodied ontological praxis. This approach not only proposes rethinking power relations and symbolically transforming caregiving spaces but also makes caregiving visible as an artistic, sensitive, and profoundly human act. In this line, Anglin et al. (2020) introduce key dimensions of aesthetic leadership, such as the aesthetic, emotional, and reflective dimensions, which promote the rehumanization of care through art and the ethical‐aesthetic development of the professional. However, this proposal still lacks a systemic conceptualization of aesthetic leadership, as well as a deep analysis of how such leadership could operate within the hierarchical, regulatory, and cultural structures of healthcare institutions. This absence of a structural perspective limits its applicability in complex organizational contexts, where leadership not only operates on an interpersonal level but also involves transforming institutional dynamics, management policies, and deeply rooted organizational cultures. The integration of these dimensions into a robust theoretical framework would consolidate aesthetic leadership not only as a theoretical alternative but also as a practical necessity for rethinking caregiving as a whole—ontologically, ethically, aesthetically, and politically.

Based on these identified coincidences, omissions, and tensions, the construction of aesthetic leadership is proposed as an epistemological, ontological, ethical, phenomenological, practical‐transformational, and political‐institutional dimension in contrast to conventional nursing leadership. Aesthetic leadership is articulated around three principles: first, the re‐signification of power relations in caregiving environments, incorporating categories such as relational poetics (understood as the ability to weave sensitive, symbolic, and ethical bonds that transform the caregiving experience into a profoundly human act), aesthetic sensitivity, and the emotional dimension as organizing principles of interactions between patients, nurses, multidisciplinary teams, affective circles, and communities; second, the transformation of clinical atmospheres and institutional spaces into aesthetically significant, emotionally inhabitable, and ontologically hospitable environments capable of generating caregiving experiences that transcend technical rationality and address suffering, vulnerability, and finitude from an aesthetics of existence; and finally, the articulation of the ethical, emotional, spiritual, and aesthetic dimensions of nursing leadership into a situated, sensitive, poetic (understood as a form of action that, far from being technical or mechanistic, cultivates meaning, beauty, symbolic expression, and the human depth of care), and emancipatory praxis that allows for re‐signifying professional practice beyond the biomedical, managerial, and technocratic model, proposing a radical rehumanization of leadership in clinical contexts.

This proposal does not imply a simple addition of the aesthetic category to existing leadership models, but rather an epistemological and ontological reconfiguration of the very concept of leadership in nursing, capable of returning centrality to sensitive knowledge, emotionality, the poetics of the environment, and the aesthetic experience of care as constitutive dimensions of contemporary nursing praxis.

## 7 Conclusions

Aesthetic leadership emerges as an innovative and profoundly transformative proposal that reconfigures the foundations of nursing leadership through a new rationality: aesthetic rationality. This is not understood as a mere ornament, but as a legitimate form of knowledge, ethical action, and relationship with the other. Aesthetic leadership in nursing is defined as an embodied and relational praxis, based on an ontology of sensitivity, an epistemology of beauty, an ethics of presence, and a politics of humanized care, integrating the aesthetic dimension as a structuring category of the act of leading in care.

This leadership model is not limited to management, organization, or motivation; it creates a world, generates meaning, transforms atmospheres, and cultivates human dignity through sensitive, expressive, and symbolic forms of the body, presence, gesture, word, silence, atmosphere, and environment.

The fundamental dimensions of aesthetic leadership in nursing are developed as follows.

### 7.1 Ontological Dimension

Aesthetic leadership is not an external function, but a way of being‐in‐the‐world‐with‐the‐other. Care is not perceived merely as a task but as a symbolic and relational experience imbued with human meaning. The aesthetic nurse leader is not just an executor of processes but an embodied presence, sensitive and meaning‐generating, who shapes relationships and environments that communicate meaning, welcome, and beauty, recognizing the other as a vulnerable and dignified subject. This leadership does not impose or dominate, but allows the other to flourish, welcoming, naming, and recognizing them. In this sense, the nurse is not defined solely by what they do, but by who they are and how they are in relation to others in the clinical encounter. This approach transcends the functional and opens the way to the poetic, understood as the ability to imbue caregiving actions with profound humanity, beauty, meaning, and hospitality in the midst of illness, fragility, pain, suffering, and/or finitude. This form of leadership can be understood from the depths, from sensitivity, and from existence.

### 7.2 Epistemological Dimension

In contrast to models focused exclusively on technical and administrative competencies, aesthetic leadership recognizes and integrates sensitive, intuitive, symbolic, and aesthetic knowledge as valid sources of clinical understanding. Aesthetic rationality is not based on the strict logic of cause and effect but on a sensitive, symbolic, and relational logic that grasps the human condition from complexity and depth. Aesthetic leadership complements and redefines technical knowledge from a sensitive and poetic rationality, not as a replacement but as an expansion of knowledge.

### 7.3 Ethical Dimension

The ethical dimension of aesthetic leadership in nursing articulates the beautiful with the good, proposing a form of leadership that not only incorporates aesthetics but leads from it. In this context, beauty is not reduced to an aesthetic‐formal criterion but acquires ethical density: it becomes a structural condition of humanized care. Caring with beauty—understood as harmony, proportion, expressivity, and openness to meaning—is equivalent to caring with dignity, recognizing in the other their irreducible value as a human being in a state of vulnerability. Beauty, in this sense, becomes a moral imperative that calls clinical leadership to create sensitive conditions that restore wounded subjectivity, existential hope, and the deep meaning of living‐in‐care. Thus, aesthetics is not an ornamental addition but a legitimate, profound, and transformative path of responsibility toward the other.

From a phenomenological and hermeneutic perspective, this clinical ethics is not reduced to universal norms or abstract rational principles but is embodied in gestures, words, silences, atmospheres, and relationships. Aesthetic leadership, as ethical praxis, also configures itself as symbolic resistance to institutional dehumanization, often marked by a functionalist, aseptic, and depersonalizing logic, and by the inability of healthcare structures to generate aesthetically meaningful experiences that dignify both the patient and the professional. In this framework, the moral value of the sensitive, atmospheric, and symbolic is recognized as ethical language capable of resisting technocratic depersonalization and restoring the deeply human character of the clinical encounter.

### 7.4 Phenomenological Dimension

Aesthetic leadership is grounded in an ontology of lived experience, in which care is configured as a way of being‐with‐the‐other in an intersubjective relationship full of meaning. It involves an attitude of openness and radical attention to phenomena as they present themselves, understanding, among other things, the pain, fragility, and hope of the other through body language, silences, and their unique way of being‐in‐the‐world. Leading aesthetically means making space for the presence of the other, not as an object of intervention but as a subject bearer of meaning, whose existence deserves to be embraced with respect, beauty, and depth.

### 7.5 Practical‐Transformational dimension

The aesthetic leadership model requires concrete praxis, transforming hospital environments into humanized spaces, training leaders sensitive to aesthetic detail, cultivating creativity as a professional competency, and creating institutional cultures that value beauty as a dimension of excellent care. It does not separate thought from action, nor aesthetics from effectiveness; it does not remain in the theoretical or philosophical realm. On the contrary, it actively transforms clinical practice, turning care into a relational art. This implies interventions that make environments and relationships experiences of care that are more beautiful, human, and dignified.

### 7.6 Political‐Institutional Dimension

Aesthetic leadership challenges dehumanizing organizational structures and proposes new forms of power based on sensitivity, hospitality, and reciprocity. It requires healthcare institutions committed to the symbolic transformation of their spaces, practices, and relationships, enabling an ethics of care grounded in the aesthetic and the human. This involves revising regulations, management models, and leadership structures that obscure the subjective experience of caring and being cared for.

In this horizon, the implications of aesthetic leadership for future research are multiple, including the exploration of phenomenological and hermeneutic methodologies to capture the aesthetics of leadership, research on the impact of spaces, music, light, or silence, among others, on the caregiving and leadership experience, the development of aesthetic competency indicators in leadership training, and the analysis of the relationship between aesthetics, organizational climate, and care quality.

Ultimately, aesthetic leadership in nursing is not limited to rethinking the practice of care, but invites us to reimagine the future of nursing from a profoundly human, ethical, and sensitive key. Thus, aesthetic leadership emerges not only as an innovative theoretical approach but as a transformative and rehumanizing force that re‐enchants care, dignifies those who practice and receive it, and opens paths toward a more conscious, compassionate, and aesthetically committed nursing practice.

## Author Contributions

Prof. Dr. Jesús Tomás Monge Moreno is the only author and the one who has elaborated all the parts of the manuscript.

## Ethics Statement

The author of this literature review article is responsible for the preparation of all sections of the manuscript. The author has no affiliations with any organization that holds direct or indirect financial interests in the subject discussed in the manuscript. The data utilized in this review were derived from previously published studies and did not involve human subjects. Consequently, informed consent was not required due to the nature of the systematic literature review. The author declares no conflicts of interest and received no funding to carry out the research reported in this manuscript. The clinical trial number is not applicable.

## Consent

All authors who subscribe to this study give their consent for it to be published.

## Conflicts of Interest

The author declares no conflicts of interest.

## Supporting information

The Supplementary.

The Supplementary.

## Data Availability

The author has nothing to report.
