# Supplementary material for: Aesthetic Leadership in Nursing: A Theoretical Proposal for Rehumanizing Care Delivery
Source: Nurs Inq. 2025 May 26;32(3):e70034. doi: 10.1111/nin.70034 (PMC12105069; doi:10.1111/nin.70034)
Supplement: Supplementary file 1 — The Supplementary. [file NIN-32-e70034-s001.docx]

**Table 1. Search strategy**

| **Search** **strategy** | |
| --- | --- |
| **S1** | ("Nursing leadership" OR "nurse leadership") AND ("Humanization of Care" OR "Patient-Centered Care" OR "humanized care" OR "compassionate care" OR "empathy") |
| **S2** | ("Clinical leadership" OR "nursing leadership") AND ("Holistic Nursing" OR "Caring" OR "humanized care") |
| **S3** | ("Nursing leadership") AND ("Patient Satisfaction" OR "patient experience" OR "empathy" OR "compassionate care") |
| **S4** | ("Ethical decision-making" OR "moral leadership" OR "Aesthetic Leadership" OR "Aesthetic nursing" OR "art of nursing" OR "nursing ethics") AND ("nursing leadership" OR "nurse leader" OR "clinical leadership") AND ("Humanization of Care" OR "patient-centered care" OR "compassionate care" OR "Caring for patients" OR "aesthetic experience") |
