# Supplementary material for: Aesthetic Leadership in Nursing: A Theoretical Proposal for Rehumanizing Care Delivery
Source: Nurs Inq. 2025 May 26;32(3):e70034. doi: 10.1111/nin.70034 (PMC12105069; doi:10.1111/nin.70034)
Supplement: Supplementary file 2 — The Supplementary. [file NIN-32-e70034-s002.docx]

*Figure 1. PRISMA Flow diagram in four levels^[[1]](#footnote-1)^*

Records identified (n = 1277):

PubMed (n = 379)

Web of Science (n = 440)

CINAHL (n = 346)

PsycInfo (n = 112)

**Identification**

Excluded records according to inclusion and exclusion criteria

(n = 651)

Records screened

(n = 626)

**Screening**

Records excluded, with reasons:

-Duplicates removed (n = 283)

-Out of aim (n = 255)

-About methodology (n = 52)

Full-text articles assessed for eligibility

(n = 36)

**Eligibility**

Studies included in review

(n = 25)

**Included**

1. *Moher, D., Liberati, A., Tetzlaff, J., Altman, D.D.G., Altman, D.D.G. and Group, P. (2009), “Preferred reporting items for systematic reviews and meta-analyses: the PRISMA statement”, Annals of Internal Medicine, Vol. 151 No. 4, pp. 264-269, doi: 10.1371/journal.pmed.1000097.* [↑](#footnote-ref-1)
